# Editorial: The impact of COVID-19 on immune system-related complications in surgical patients

**DOI:** 10.3389/fsurg.2023.1132752

**Published:** 2023-01-30

**Authors:** Philip F. Stahel, Sebastian Weckbach, Markus S. Huber-Lang, Vincent P. Stahel, Scott R. Barnum

**Affiliations:** ^1^ Department of Surgery, East Carolina University, Brody School of Medicine, Greenville, NC, United States; ^2^ Department of Specialty Medicine, Rocky Vista University, College of Osteopathic Medicine, Parker, CO, United States; ^3^ Department of Spine Surgery, NeuroSpine Zürich, Zürich, Switzerland; ^4^ Institute for Clinical and Experimental Trauma-Immunology, University Hospital Ulm, Ulm, Germany; ^5^ University of Colorado, Boulder, CO, United States; ^6^ CNine Biosolutions LLC, Birmingham, AL, United States

**Keywords:** COVID-associated coagulopathy, fibrinolytic shutdown, hyperinflammation, innate immunity, surgical complications

**Editorial on the Research Topic** The impact of COVID-19 on immune system-related complications in surgical patients

A key lesson learned from the coronavirus disease 2019 (COVID-19) pandemic is reflected by the finding that patients with severe acute respiratory syndrome coronavirus-2 (SARS-CoV-2) infection have a significantly increased mortality and complication rates after surgery, compared to surgical patients who are SARS-CoV-2 negative (1–3). This phenomenon has been primarily attributed to respiratory failure from SARS-CoV-2 pneumonia and acute respiratory distress syndrome (ARDS) subsequent to mechanical ventilation for general anesthesia during surgical procedures (4). In addition to the well-known pulmonary complications, young patients with COVID-19 are particularly susceptible to adverse effects originating from a dysfunction of the innate immune system (5–7). This includes the release of a “cytokine storm” and activation of the complement system which promotes a systemic environment of hyperinflammation and hypercoagulability (8–15). These pathophysiological changes may contribute to the increased perioperative risk of thromboembolic complications and the high postoperative mortality observed in surgical patients with COVID-19 (16–20). The current special edition in *Frontiers in Surgery* was designed to improve the understanding of the immune-mediated pathophysiological events that lead to adverse outcomes in the vulnerable population of COVID-19 patients undergoing surgical procedures.

**Figure 1:**
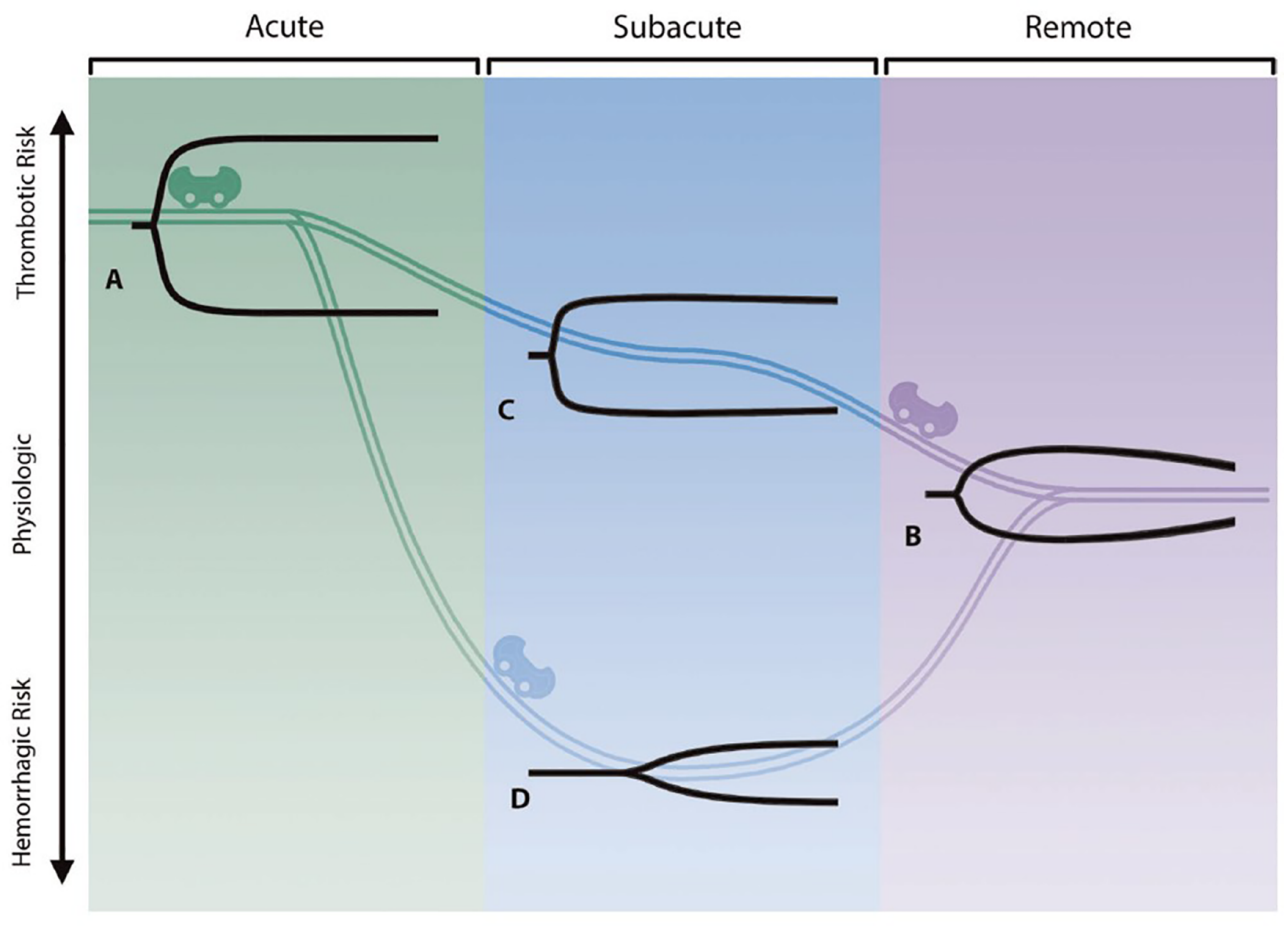
The three stages constituting the “rollercoaster” phenomenon of COVID-associated coagulopathy, as determined by thromboelastography (TEG). See original publication for detailed explanation of the content (21).

An authoritative review from an international consensus group coherently summarized the current state-of-the-art in the field as it pertains to the pathogenesis of SARS-CoV-2-mediated immune-thrombotic complications after surgery Bunch et al. The authors introduced the new entity of “COVID-associated coagulopathy” and explained the underlying hypothesis related to SARS-CoV-2-induced endothelitis as the root cause of an imbalance between fibrinolysis and fibrinolytic shutdown Bunch et al. The clinical implications pertain to adjudicating the timing of elective surgical procedures in COVID-19 patients and to monitor the “rollercoaster” stages of COVID-associated coagulopathy by point-of-care-guided viscoelastic hemostatic assays, such as thromboelastography (Figure 1). The authors furthermore provided specific clinical recommendations for perioperative anticoagulation in COVID-19 patients for a variety of surgical subspecialties, including general surgery, cardiothoracic and vascular surgery, reconstructive surgery, obstetrics, orthopedics, and neurosurgery Bunch et al. A retrospective observational cohort study from the Middle East confirmed the notion of an increased perioperative mortality in COVID-19 patients undergoing orthopedic surgery and spine procedures during the early surges of the pandemic in 2020 with a demonstrated protective effect of SARS-CoV-2 vaccinations in 2021 Kim et al. These findings furthermore provided additional support to the safety and efficacy of mRNA vaccines in the selected cohort of surgical COVID-19 patients (21).

Another observational study from a tertiary referral center in Taiwan assessed patient-reported outcome measures (PROMs) after spine surgery and determined that patients had worse subjective postoperative outcomes during the COVID-19 pandemic Lin et al. While the root cause of the decrease in PROMs remains speculative, the authors recommend consideration for psychological support for surgical patients during the pandemic to improve postoperative outcomes Lin et al. A similar publication from Germany reported unchanged infectious complication rates after spinal surgery during the COVID-19 pandemic from April 2020 to June 2021, when compared to a historic pre-pandemic cohort of spine surgery patients managed at the same institution from January 2019 until March 2020 (22). The authors attributed the lack of decreased surgical site infections, in spite of the COVID-19 preventative measures, to higher rates of emergency surgical spine procedures during the pandemic (22). The special edition concludes with a case report on three patients with COVID-19 who sustained severe osteomyelitis of the jaw as a rare but severe infectious complication Kvolik Pavic et al. The authors hypothesized that the SARS-CoV-2-induced immune dysfunction and microvascular changes contributed to the increased vulnerability for severe infection in patients with COVID-19 Kvolik Pavic et al.

In summary, the profound immunological derangements induced by SARS-CoV-2 infection significantly increase the risk of perioperative complications and adverse outcomes in COVID-19 patients undergoing surgical procedures. Delaying elective surgery for 6 weeks after infection has been proposed by surgical societies as a strategy to prevent adverse outcomes. However, this conservative strategy represents a double-edged sword due to the risk associated with depriving patients from access to timely surgical care (23). There are currently multiple anti-inflammatory pharmacological agents available to attenuate the SARS-CoV-2-induced immune system derangements, including complement inhibitors (8, 24, 25) and monoclonal antibodies to pro-inflammatory cytokines, such as interleukin (IL)-1, IL-6, IL-22 or IL-33 (26–28). Now at three years into the pandemic, the lessons learned from the COVID-related immunopathology will allow for more specifically targeted treatment modalities to inhibit SARS-CoV-2-induced hyperinflammation and the related immuno-thrombotic sequelae responsible for the high complication rates and increased mortality in surgical COVID-19 patients.
